# Applied Behavior Analysis in the Crosshairs: Neurodiversity, the Intact Mind, and Autism Politics

**DOI:** 10.1007/s40614-025-00439-3

**Published:** 2025-03-03

**Authors:** Amy S. F. Lutz

**Affiliations:** History and Sociology of Science Department, University of Pennsylvania, 249 South 36th Street, Philadelphia, PA 19104 USA

**Keywords:** Autism, Intact mind, ABA, Neurodiversity

## Abstract

Recent attacks on applied behavior analysis (ABA) by neurodiversity advocates share a common theme with opposition to other supports, such as subminimum wage vocational programs and congregate residential settings: the intact mind assumption, which maintains that even profoundly autistic people have typical intelligence, even if they present as severely cognitively impaired. This article examines the history of the intact mind assumption, which was largely shaped by psychoanalytic theory in the mid-20^th^ century, as well as its impact on contemporary disability policy and practice.

Applied behavior analysis (ABA) has never been without its critics. Precursor interventions based on the principles of operant conditioning, such as that of O. Ivar Lovaas, were castigated for the routine use of punishment, including slaps and shocks. Discrete trial training, one of the earliest forms of ABA, has been accused of being tediously repetitious and non-generalizable (Garey, 2024). But these objections pale in comparison to our current moment, in which ABA is categorically and indiscriminately attacked, frequently on social media, as “dehumanizing” (Liberation Caucus, 2021), “torture” (Isabella Scarlett, 2024), and a “human rights violation” (A Happy Place, 2024). There is even a #BanABA movement—which makes me wonder if, should it succeed, my husband and I would have to throw away the laminated list of steps stuck to the shower wall for my 25-year-old profoundly autistic son to follow? Or stop reinforcing his pausing at the curb instead of dashing into traffic? Or give up searching for a replacement behavior for the head-slapping that Jonah engages in when he’s agitated, which is causing him to develop cauliflower ear? Particularly perplexing about this ferocious anti-ABA rhetoric is how it completely ignores the variety of interventions in the ABA arsenal—most of which, I suspect, even the staunchest anti-ABA activists would find completely unobjectionable.

Why, then, are they so angry? Several claims are frequently made: that ABA makes autistic people unduly compliant (Kaylene, 2024); that it causes PTSD symptoms, including nightmares and flashbacks (Kupferstein, 2018); that its goal is the elimination of benign behaviors, like stimming, to make autistic people appear more “normal” (Kaylene, 2024) (although, as I’ve written elsewhere—in an article whose title pretty much sums up the view from the profound end of the autism spectrum, “Who Cares About Eye Contact or Hand Flapping?” (Lutz, 2019)—the parents I know are working instead on key goals such as physical safety, communication, toileting, and basic life skills).

There’s another reason that I’ll focus on in this article, because it ties the war against ABA to other, seemingly unrelated, policy debates across the autism community, such as current efforts to educate all autistic kids in inclusive classrooms, eliminate the subminimum wage, close congregate residential settings, and replace guardianship with more informal types of support. These fights are all championed by neurodiversity advocates, who consider autism an identity, like homosexuality, rather than a disorder, and who push for narratives that emphasize autistic strengths, rather than deficits. Neurodiversity advocates—who are, themselves, overwhelmingly mildly affected autistic adults—simply don’t accept that many profoundly autistic people need qualitatively, not just quantitatively, different services to achieve maximal outcomes than those required by individuals at the other end of the spectrum. As one autistic adult posted on X (formerly Twitter) about alternatives to ABA: “As if there’s a separate formula for autistic kids, instead of just your regular love & support from a parent. Autistic kids are children. They don’t need an alternative” (AutSciPerson, 2021).

Implicit in this position is what I call the intact mind assumption—the belief that, hidden deep inside all autistic people, no matter how impaired they may present, is a typical or even genius intellect. Once you know to look for it, it becomes obvious that this idea underpins the entire neurodiversity agenda, from the opposition to specialized, disability-specific settings and interventions just described, to their support for facilitated communication, a thoroughly discredited, pseudoscientific tool through which profoundly autistic and otherwise intellectually disabled people allegedly spell out sophisticated, articulate messages on a letterboard held by a nondisabled facilitator. And the attack against ABA becomes part of a larger landscape in which the severely cognitively disabled are marginalized, their intense needs eclipsed by the preferences of a population that would never qualify for state- or insurance-funded supports anyway.

The intact mind assumption did not originate with the neurodiversity movement. As I’ll demonstrate below, it has been part of autism discourse since Leo Kanner proposed the diagnosis in 1943. But what the neurodiversity movement has done so successfully is weaponize identity politics against its critics, casting them as “ableist.” Today, if you describe profoundly autistic people who have severe cognitive impairment, minimal functional skills, and extraordinarily challenging behaviors such as elopement, fecal smearing, aggression, or self-injurious behaviors—even with the goal of developing interventions for these extraordinarily challenging and life-limiting symptoms—you will likely be publicly accused of infantilizing, disparaging, or discriminating against the disabled.

## The History of the Intact Mind

Today, we classify autism as a developmental disability, alongside other conditions such as Down syndrome, cerebral palsy, and spina bifida. But in 1943, autism was considered to be closely related (for some physicians, indistinguishable from) childhood schizophrenia. This era was the heyday of psychoanalysis in the United States, the fundamental premise of which was that psychopathology was caused by early childhood trauma—which largely meant bad parenting (and bad mothering in particular). When Kanner blamed his young patients’ symptoms on the “emotional refrigeration” they experienced at the hands of frigid mothers who offered “mechanized service of the kind which is rendered by an overconscientious gasoline station attendant” (Kanner, 1954), his etiological theory was perfectly aligned with those of prominent psychoanalysts like Beata Rank—who claimed that the kids she treated all “suffered gross emotional deprivation” at the hands of a “dangerous mother” who was “emotionally disturbed,” “narcissistic,” and “immature” (Rank, 1949)—and Frieda Fromm-Reichmann, who in 1948 famously coined the term “schizophrenogenic mother” to describe women who created psychopathology through their “severe early warp and rejection” of their children (Fromm-Reichmann, 1948).

Clara Claiborne Park wrote the very first autism parent memoir in 1967 to push back against such parent-blaming narratives. As she pointed out in *The Siege,* Park had treated her autistic (and youngest) daughter exactly the same as she had her three older children, so if autism was caused by so-called “refrigerator mothers,” then why weren’t her other kids affected?

Autism parents were tremendously grateful for Park’s defense: *The Siege* “made waves throughout America” (Feinstein, 2010), was translated into Spanish, French, German, Japanese, and several other languages (Vicedo, 2021), and is still read today. But the book’s most enduring legacy is one Park almost certainly never anticipated. As much as she challenged the experts who blamed her for her daughter’s autism, she did so in psychoanalytic language and imagery that fixed the intact mind in public conceptions of autism going forward.

It’s right there in the title, *The Siege,* which also provides the guiding metaphor throughout the book, from the dedication—“To those behind walls, and to all their besiegers”—to this reflection in the closing chapter: “Of all the things that Elly has given, the most precious is this faith, a faith experience has almost transformed into certain knowledge: that inside the strongest citadel he can construct, the human being awaits his besieger” (Park, 1967). This last sentence is indistinguishable from those written by prominent psychoanalysts, right down to the architectural metaphors—such as Margaret Mahler, who described “primary autism” as a condition in which “there is a deanimated, frozen wall between the subject and the human object” (Mahler et al., 1975), or Mira Rothenberg, who “wanted to help [her patients] to help themselves creep out of their terrifying fortresses” (Rothenberg & Page, 1960).

Also lifted straight from the psychoanalytic playbook is the implication that children *choose* to retreat into autism. For Park, this is a defining feature of the disorder—in her first description of it, she describes the paradigmatic affected child as “locked in himself, secure and satisfied in his repetitive activities, he will not use [his intelligence], or even display it for testing” (Park, 1967). And her chapter names—including “Willed Weakness,” “Willed Blindness,” “Willed Deafness,” and “Willed Isolation”—could have been crafted by Louise Despert, who emphasized, with italics, autistic children’s “*determination not to communicate*” (Despert, 1965) or Virginia Axline’s evocation of the “wall [her patient] has built so sturdily around himself” (Axline, 1964).

It would not be long before medical thinking around autism shifted, as psychiatry abandoned psychoanalysis in the 1970s and embraced a more biological framework that focused on inborn causes of psychopathology. British psychiatrist Michael Rutter had rejected the intact mind narrative as early as 1965: “It has sometimes been thought that all autistic children are ‘really’ of normal intelligence, and that intellectual brilliance always exists beneath the autistic surface of failures to speak, learn and form relationships. Regrettably, this is not the case” (Rutter, 1965).

This realignment should theoretically have left physicians and families on the same side. But the new medical model of autism etiology carried a decidedly unwelcome prognosis. The one positive takeaway from the “refrigerator mother” theory is that it offered more hope for recovery: if parents had done something to their previously normal children to cause autism, then it made sense that something else could be done to change them back. So parents in particular were reluctant to abandon the intact mind assumption, which persists in autism parent memoirs from *The Siege* all the way to the present. Just consider, for example, the titles of some 21^st^-century books: *There’s a Boy in Here,* by Judy and Sean Barron; *I Know You’re in There,* by Marcia Hinds; *I’m So Glad You Found Me in Here,* by Matthew and Nancy Hobson. Summarizes disability scholar Stuart Murray, “This central idea here of a space that is ‘somewhere behind,’ or of being ‘locked in’ . . . is, of course, a virtual orthodoxy in narrative depictions of autism” (Murray, 2008).

## Profound Autism in the Real World

It’s completely understandable why so many parents are reluctant to give up the dream of the intact mind. Are there any larger stakes in the parenthood game? The intact mind is the lifeline to the completely ordinary future all parents want for their children. A life of virtually limitless options—from the big decisions, like where to live or work, to the seemingly insignificant inclinations to order a pizza or even just open the door and take a walk. Accepting a child’s complete and lifelong dependence can be paralyzing. As Virginia Breen—author of a memoir about how her daughter’s intact mind was supposedly tapped through facilitated communication—reflects, “In Dante’s *Inferno*, above the gates of hell a sign reads, ‘Abandon all hope.’ I cannot think of a more apt description of hell—a place without hope. Yet the burden of autism can push us to live in that very place. . . . Somehow, autism moms have to find a way to cling to hope with a tenacity that is stronger than autism’s grip on our children. But how?” (Bonker & Breen, 2011).

Still, most parents eventually come to accept and love their profoundly autistic children as they are, not as they wish they were. No matter where families are on this journey, ABA providers and other professionals who work with this population—including psychologists, psychiatrists, occupational therapists, speech/language pathologists, etc.—are critical allies in the fight to ensure that profoundly autistic children and adults are appropriately supported across their lifespans. It is important to note that this fight isn’t against neurodiversity activists. Responding directly to ideologues blasting ABA as torture on social media, for example, is a fruitless pursuit that invites only more venom.

Instead, we need to make this population legible to policy makers. That means using accurate and meaningful descriptors, even as neurodiversity advocates publish language guides in prominent journals to try and control the very words we use to describe our profoundly autistic loved ones, clients, patients, and research subjects—guides that essentially proscribe vocabulary most commonly attached to those on the most severe end of the spectrum, including functioning labels of any kind as well as the terms “disorder,” “severe,” “challenging behavior,” even “treatment” (Bottema-Beutel et al., 2021). Although such language policers can be very aggressive (particularly on social media), it’s important to note that the growing consensus behind the term “profound autism” itself—first proposed by the Lancet Commission in 2021 and met with vigorous opposition from neurodiversity advocates—suggests that most stakeholders in the autism community actually support the need for “a full semantic toolbox” (Singer et al., 2022) to address autism’s most pressing challenges. Even the CDC—never accused of being on the cutting edge of anything—is using the term now, publishing the first official prevalence figures for this specific subpopulation in 2023 (27% of the most recent cohort of autistic 8-year-olds met their criteria, which include limited or no communication and/or IQ below 50; Hughes et al., 2023).

It also means insisting on evidence-based interventions—a directive that converges nicely with the emphasis on data collection in ABA. But again, this isn’t just about defending ABA. In a broad sense, it’s about ensuring that bureaucrats and elected officials at the state and federal levels set disability policy that is supported by research, not ideology. As I write this in December 2024, the Department of Labor has just issued a Notice of Proposed Rulemaking that, if finalized, would eliminate 14(c), the regulation that allows some employers (virtually all nonprofit disability service providers) to pay some cognitively impaired employees a subminimum wage based on specified metrics of productivity. Central to neurodiversity opposition to 14(c) is the assertion that all autistic and otherwise intellectually disabled people are capable of working a minimum-wage job, given the proper supports. But this is just the intact mind in action. What the limited research from states that have already eliminated their 14(c) programs demonstrates time and again is that, post-closure, most former participants end up sitting at home (see Center for Health Care Strategies, 2012; Milken Institute School of Public Health, 2015; Spreat & Conroy, 2015). It’s extraordinarily ironic: we’ve built a service system for the intellectually disabled that assumes they aren’t really intellectually disabled. No wonder it’s failing so many families. It’s time to re-center our policy discourse instead on the needs and preferences of the individuals with impaired minds who depend upon it most.

## Data Availability

No data was used in this commentary.

## References

[CR1] A Happy Place. (2024). “ABA is NOT the Answer!” https://ahappyplace.co.za/learn-more/f/aba-is-not-the-answer. Accessed 25 Jan 2025.

[CR2] AutSciPerson. (2021). [tweet]. https://twitter.com/AutSciPerson/status/1375100094316445696. Accessed 25 Jan 2025.

[CR3] Axline, V. M. (1964). *Dibs in search of self*. Ballantine Books.

[CR4] Bonker, E. M., & Breen, V. G. (2011). *I am in here: The journey of a child with autism who cannot speak but finds her voice*. Revell.

[CR5] Bottema-Beutel, K., Kapp, S. K., Lester, J. N., et al. (2021). Avoiding ableist language: Suggestions for autism researchers. *Autism in Adulthood,**3*(1), 18–29. 10.1089/aut.2020.0014 36601265 10.1089/aut.2020.0014 PMC8992888

[CR6] Center for Health Care Strategies. (2012). Trends and challenges in publicly-financed care for individuals with intellectual and developmental disabilities. http://www.chcs.org/media/IDD_Service_Delivery_Systems_082812.pdf. Accessed 25 Jan 2025.

[CR7] Despert, J. L. (1965). *The emotionally disturbed child: Then and now*. Robert Brunner.

[CR8] Feinstein, A. (2010). *A history of autism: Conversations with the pioneers*. John Wiley & Sons.

[CR9] Fromm-Reichmann, F. (1948). Notes on the development of treatment of schizophrenics by psychoanalytic psychotherapy. *Psychiatry,**11*(3), 263–273. 18889231 10.1080/00332747.1948.11022688

[CR10] Garey, J. (2024). *The controversy around ABA*. Child Mind Institute. https://childmind.org/article/controvers.y-around-applied-behavior-analysis/. Accessed 25 Jan 2025.

[CR11] Hughes, M. M., Shaw, K. A., DiRienzo, M., Durkin, M. S., Esler, A., Hall-Lande, Wiggins, J., Zahorodny, W., Singer, A., & Maenner, M. J. (2023). The prevalence and characteristics of children with profound autism, 15 sites, United States, 2000–2016. *Public Health Reports,**138*(6), 971–980. https://pubmed.ncbi.nlm.nih.gov/37074176/. Accessed 25 Jan 2025. 10.1177/00333549231163551 PMC10576490 37074176

[CR12] Isabella Scarlett. (2024) [tweet], https://x.com/IsabellaScar2/status/1775190354318197025. Accessed 25 Jan 2025.

[CR13] Kanner, L. (1954). To what extent is early infantile autism determined by constitutional inadequacies? *Association for Research in Nervous & Mental Disease,**33*(1), 378–385. 13246089

[CR14] Kaylene. (2024). *5 important reasons even “New ABA” is problematic*. Autistic Mama. https://autisticmama.com/even-new-aba-is-problematic/. Accessed 25 Jan 2025.

[CR15] Kupferstein, H. (2018). Evidence of increased PTSD symptoms in autistics exposed to applied behavior analysis. *Advances in Autism,**4*(1), 19–29.

[CR16] Liberation Caucus. (2021) [tweet]. https://x.com/LPLiberation/status/1412932635194757127. Accessed 25 Jan 2025.

[CR17] Lutz, A. S. F. (2019). Who cares about eye contact or hand flapping. *Psychology Today*. https://www.psychologytoday.com/us/blog/inspectrum/201910/who-cares-about-eye-contact-or-hand-flapping. Accessed 25 Jan 2025.

[CR18] Lutz, A. S. F. (2023). Chasing the intact mind: How the severely autistic and intellectually disabled were excluded from the debates that affect them most. Oxford University Press.

[CR19] Mahler, M. S., Pine, F., & Bergman, A. (1975). *the psychological birth of the human infant*. Basic Books.

[CR20] Milken Institute School of Public Health. (2015). Transitions: A case study of the conversion from sheltered workshops to integrated employment in Maine. Department of Health Policy & Management, George Washington University https://docplayer.net/33593240-Transitions-a-case-study-of-the-conversion-from-sheltered-workshops-to-integrated-employment-in-maine.html. Accessed 25 Jan 2025.

[CR21] Murray, S. (2008). *Representing autism: Culture, narrative, fascination*. Liverpool University Press.

[CR22] Park, C. C. (1967). *The siege: A family’s journey into the world of an autistic child*. Harcourt, Brace &World.

[CR23] Rank, B. (1949). Adaptation of the psychoanalytic technique for the treatment of young children with atypical development. *American Journal of Orthopsychiatry,**19*(1), 130–139. 18106791 10.1111/j.1939-0025.1949.tb06567.x

[CR24] Rothenberg, M., & Page, H. (1960). The rebirth of Jonny. *Harper’s Magazine,**220*(1317), 57–66.

[CR25] Rutter, M. (1965). Medical aspects of the education of psychotic (autistic) children. In P. T. B. Weston (Ed.), *some approaches to teaching autistic children: A collection of papers* (pp. 61–74). Pergamon Press.

[CR26] Singer, A., Lutz, A., Escher, J., & Halladay, A. (2022). A full semantic toolbox is essential for autism research and practice to thrive. *Autism Research,**16*(3), 497–601. 10.1002/aur.2876 36508163 10.1002/aur.2876

[CR27] Spreat, S., & Conroy, J. W. (2015). Longitudinal investigation of vocational engagement. *Journal of Policy & Practice in Intellectual Disabilities,**12*(4), 266–271.

[CR28] Vicedo, M. (2021). *Intelligent love: The story of Clara Park, her autistic daughter, and the myth of the refrigerator mother*. Beacon Press.

